# Neuroprotective Effect of Syringic Acid by Modulation of Oxidative Stress and Mitochondrial Mass in Diabetic Rats

**DOI:** 10.1155/2020/8297984

**Published:** 2020-12-04

**Authors:** Marzieh Rashedinia, Mahshid Alimohammadi, Nazgol Shalfroushan, Mohammad Javad Khoshnoud, Mitra Mansourian, Negar Azarpira, Zahra Sabahi

**Affiliations:** ^1^Medicinal Plants Processing Research Center, Shiraz University of Medical Sciences, Shiraz, Iran; ^2^Department of Pharmacology and Toxicology, School of Pharmacy, Shiraz University of Medical Sciences, Shiraz, Iran; ^3^Food and Supplements Research Center, Shiraz University of Medical Sciences, Shiraz, Iran; ^4^Transplant Research Center, School of Medicine, Shiraz University of Medical Sciences, Shiraz, Iran

## Abstract

Diabetes is a metabolic complaint associated with oxidative stress and dysfunction of mitochondria. One of the most common complications of diabetes mellitus is neuropathy. This study evaluated the possible neuroprotective effects of syringic acid (SYR), a natural polyphenolic derivative of benzoic acid, on oxidative damage and mitochondria in the brain, spinal cord, and sciatic nerve of streptozotocin-induced diabetic rats. Different groups of rats including normal control, diabetics (induced by streptozotocin), diabetic groups treated with 25, 50, and 100 mg/kg of SYR, and non-diabetic group treated with only 100 mg/kg of SYR were treated for 6 weeks. Learning and memory function, physical coordination, and acetylcholinesterase (AChE) and antioxidant indexes, as well as mRNA expression of mitochondrial biogenesis, were measured in the brain, spinal cord, and sciatic nerves. Diabetic rats treated with 100 mg/kg SYR exhibited significantly improved learning, memory, and movement deficiency (*p* < 0.05). SYR 100 mg/kg also significantly upregulated the brain mRNA expression of PGC-1*α* and NRF-1, the key regulators of energy metabolism, oxidative phosphorylation, and mitochondrial biogenesis. In addition, SYR 100 mg/kg and SYR 50 mg/kg increased the mtDNA/nDNA ratio in the brain and the spinal cord of diabetic rats, respectively (*p* < 0.05). SYR attenuated the lipid peroxidation in all the tissues, but not significant effects were observed on GSH, AChE, catalase, and superoxide dismutase activity. In all the tests, nonsignificant differences were observed between the control and SYR 100 mg/kg groups. Moreover, SYR reduced inflammation and demyelination in sciatic nerves. This is the first study to reveal the regulation of mitochondrial biogenesis and energy metabolism by SYR, beyond its antioxidant role in the diabetic rats' brain and spinal tissues.

## 1. Introduction

Diabetes mellitus refers to a metabolic disorder involving the insufficiency of insulin secretion and/or insulin resistance. Approximately 82 million people suffer from this disease worldwide, and this number may double by 2030 [1]. The prevalence of this problem is projected to increase from 2.8% in 2000 to 4.4% in 2030 [2].

The uncontrolled chronic hyperglycemia of diabetes leads to diabetic complications in various organs such as retinopathy, nephropathy, cardiomyopathy, and neuropathy [1]. A diabetic's hyperglycemia also results in the generation of free radicals because of the autoxidation of glucose and glycosylation of proteins. Constant production of reactive oxygen species (ROS) and reactive nitrogen species (RNS), as well as the reduction in the capacity of antioxidants, leads to oxidative stress, which is the main cause of insulin resistance [2]. Diabetic neuropathy is a prevalent disorder affecting both central (CNS) and peripheral nervous systems (PNS) [3]. Diabetic peripheral neuropathy (DPN) is the most common neuropathic syndrome, often following a chain of changes in the PNS as well as the CNS. Moreover, central neuropathy is characterized by Alzheimer's disease-like features in the brain which increase the risk of dementia in the diabetic patients [4]. While the pathologic mechanisms leading to damage of CNS and PNS are not well understood, one of the proposed mechanisms is the contribution of impaired insulin support [5]. Studies have shown that increased glucose concentration increases the level of glutamate, as an excitatory neurotransmitter, which leads to a cognitive dysfunction by causing neuronal damage in the CNS [6]. Furthermore, insulin resistance which causes hyperglycemic conditions is associated with mitochondrial abnormalities and increases the reactive oxygen species (ROS) production in the electron transport chain and also a deficiency in antioxidant defence system [7]. The generation of free radicals induces tissue injury in the peripheral nerve of experimental diabetic rats [8]. Chronic hyperglycemia-induced oxidative stress leads to impaired mitochondrial function and apoptosis, and it initiates damage of dorsal root ganglion and axons of sensory neurons. In addition to increasing the mitochondrial fission and biogenesis, it reduces motor and sensory conduction velocities [9, 10]. Moreover, it was found that oxidative stress and its consequent processes not only have adverse impacts on the brain but are also related to spinal cord injury [11]. Nowadays, public and scientific attention has been drawn to using phytochemicals to control human diseases. Studies have indicated that antioxidant compounds from medicinal plant sources such as polyphenols have a mitigating impact on oxidative stress and mitochondrial protective effects and directly interact with mitochondrial biomolecules [12, 13].

The focus of this study is on antidiabetic effects of syringic acid (SYR). SYR is a phenolic acid derivative of hydroxybenzoic acid and is widely present in pumpkin, olives, grapes, acai palm, and the leaves of *Alpinia calcarata* Roscoe [14]. SYR is known as a natural antioxidant with free radical scavenging effects and potential biological activities such as antimicrobial, anticancer, anti-inflammation, antidiabetic, and cardioneuro protective effects [15, 16]. The therapeutic properties of SYR are associated with the presence of methoxy groups onto the aromatic ring in its structure. It is able to scavenge free radicals, regulate enzyme activity, and diverse transcription factors involved in diabetes, inflammation, angiogenesis, and cancer [15]. Furthermore, SYR had neuroprotective effects on oxidative stress and axonal degeneration in rodent sciatic nerves after ischemia-reperfusion injury [17]. Also, in our previous studies, we observed that SYR improved hepatic complications [18] and nephropathy in diabetic rats [19].

Since the complication of diabetes has been known as an oxidative stress disorder, natural antioxidants can have beneficial effects against secondary complications of diabetes such as neuropathy. SYR is able to eliminate the free radicals and tend to modulate various transcriptional factors in neuroprotective and diabetic animal models [15, 20, 21]. This study was designed to evaluate the effects of SYR administration on learning, memory, and oxidative status and mitochondrial function through mRNA expression of the key regulators of energy metabolism and mitochondrial biogenesis in the diabetic rat brain, spinal cord, and sciatic tissues.

## 2. Materials and Methods

### 2.1. Chemicals

All the chemicals that were used in this experiment were purchased from Sigma-Aldrich (St. Louis, USA) or Merck Company (Darmstadt, Germany). The highest commercially available grade was used.

### 2.2. Animals

Male Sprague-Dawley rats (220-240 g weight) were obtained from the Centre of Comparative and Experimental Medicine of Shiraz University of Medical Sciences (Shiraz, Iran). The animals were housed in cages at a temperature of 25 ± 3°C and 12 h light/dark cycle. All rats had access to water and food ad libitum. All animal procedures were performed in accordance with the Guide for the Care and Use of Laboratory Animals and approved by the local ethics committee (Permit # IR.SUMS.REC.1397.183, Shiraz University of Medical Sciences, Shiraz, Iran). Diabetes mellitus was experimentally induced in animals by injecting 60 mg/kg of streptozotocin (STZ) (freshly dissolved in citrate buffer 0.1 M, pH 4) intraperitoneally after fasting for 12 h. Nondiabetic control animals received an equivalent volume of saline by intraperitoneal injection. One week after the STZ treatment, the level of glucose was measured in the blood samples taken from the tail vein using a glucometer (Gluco Lab, Korea). Rats with blood glucose levels more than 300 mg/dl were included in the study [22].

### 2.3. Study Design

Thirty-six animals were randomly divided into six groups (*n* = 6): group I: nondiabetic control, group II: diabetes control (STZ-induced), group III: diabetic+SYR (25 mg/kg), group IV: diabetic+SYR (50 mg/kg), group V: diabetic+SYR (100 mg/kg), and group VI: nondiabetic SYR (100 mg/kg). SYR was administered once per day orally using an intragastric gavage for a period of six weeks. Nondiabetic and diabetic control groups were treated with saline. After 6 weeks of treatment, behavioural tests were performed, and then, the animals were sacrificed by cervical decapitation. The brain, spinal cord, and sciatic tissues were removed and washed in ice-cold saline; separated tissue fragments were weighed and homogenized in cold phosphate buffer (0.1 M, pH 7.4). Homogeneous tissues were aliquoted for biochemical analysis and RNA and DNA extraction and frozen at -70°C until use. The sciatic nerve of each rat was fixed in 10% formalin, embedded in paraffin, and cut to 5 *μ*m thickness. The tissue slices were stained using hematoxylin and eosin (H&E) and Luxol fast blue (LFB) techniques.

### 2.4. Behavioural Tests

#### 2.4.1. Passive Avoidance Test (Shuttle Box)

The shuttle box consisted of two equal-sized light and dark compartments. The two compartments were separated by a guillotine door. The floor of the dark compartment consisted of a stainless steel shock grid floor. Electric shocks were delivered to the grid floor with a stimulator (50 Hz, 1 sec, 1.5 mA intensity). This test has two phases, learning and memory evaluation. If the rat has successfully completed the learning phase, it avoids entering the dark compartment or enters with a delay in the memory phase. The delay in entering the dark compartment, known as step-through latency (STL), was recorded to a maximum of 300 seconds. Short latencies indicate poor retention compared to significantly longer latencies [23].

#### 2.4.2. Motor Performance Test

The rotarod test is widely used for evaluating motor coordination, balance, and locomotor activity in rodents. The apparatus consists of a circular rod turning at a constant or increasing speed. A rotarod apparatus with programmed timers and falling sensors was used in this survey. The animals were placed on a rod after being pretrained on the rotarod machine. The rats were tested at using a fixed-speed rotarod procedure. During the testing period, animals were tested at 10 rpm speed for a maximum of 60 s. The rats experienced the test three times at the determined speed with an interval of 20 min between each test. The latency to fall was recorded in each experiment [24].

### 2.5. Biochemical and Molecular Tests

#### 2.5.1. Determination of Glutathione (GSH)

The glutathione contents of the brain and spinal cord were assessed using the Ellman reagent. Equal amounts of homogenized tissues and 10% *w*/*v* trichloroacetic acid were mixed. After centrifugation, 500 *μ*l of supernatant was mixed with reaction buffer containing 3 ml of phosphate buffer (0.3 M, pH 8.9) and 500 *μ*l of dithiobisnitrobenzoic acid (DTNB) (0.01 M). The absorbance of yellow colour was read at 412 nm using BioTek ELX800 ELISA Reader, USA [25].

#### 2.5.2. Acetylcholinesterase (AChE) Activity

Homogenized brain tissue was centrifuged for 10 minutes at 5000 rpm, and then, the supernatant was used to measure AChE activity according to the Ellman spectrophotometric method [26]. In this method, AChE hydrolyzes ACh to produce thiocholine and acetate. The thiocholine reduces the DTNB to nitrobenzoate (TNB) anions, a yellow-coloured compound, which has an absorbance at 405 nm. The increase in absorbance at 412 is proportional to the enzyme activity. The activity of the enzyme was calculated using the extinction coefficient of TNB anions.

#### 2.5.3. Lipid Peroxidation Measurement

Lipid peroxidation in the brain, spinal cord, and sciatic nerve was assessed using thiobarbituric acid reactive substances (TBARS) and malondialdehyde (MDA). The reaction mixture consisted of 500 *μ*l of 10% homogenized tissue, 250 *μ*l of HCl (0.5 N), and 500 *μ*l of 0.6% TBA. This mixture was vortexed and then heated in boiling water for 45 min. After cooling and centrifuging (6000 rpm, 10 min), the absorbance of the supernatant was measured at 540 nm using an automated plate reader. The level of MDA was reported as nmol/g tissue [27].

#### 2.5.4. Antioxidant Enzyme Activity

Catalase activity was determined according to the method described previously [28]. Briefly, 60 *μ*l of the tissue homogenate supernatant was incubated in 300 *μ*l substrate (130 mmol/kg hydrogen peroxide in 60 mmol PBS, pH 7.4) for 3 minutes. The reaction was stopped by adding 300 *μ*l of 32.4 mmol ammonium molybdate, and the absorbance was measured at 405 nm against the blank. The enzyme activity was calculated and expressed as U/l. Superoxide dismutase (SOD) activity in the brain tissue was measured using a commercial kit (Nasdox, Navand Salamat, Iran) following the instructions of the manufacturer, based on the pyrogallol autoxidation method [29].

#### 2.5.5. Mitochondrial Biogenesis Indices of the mRNA Expression Level

The fresh homogeneous brain was subjected to RNA extraction. Total RNA was isolated using the Pars Tous kit (Mashhad, Iran), quantified (NanoDrop), and standardised. The complementary DNA (cDNA) synthesis was performed (Easy cDNA Synthesis Kit; Pars Tous, Iran). Gene expression was determined using a SYBR Green PCR kit (Amplicon) according to the manufacturer's protocol. 18s rRNA was used as an internal control for normalization [30]. The sequences of primers used in real-time PCR were as follows: TFAM: 5′-GAAAGCACAAATCAAGAGGAG-3′ and 5′-CTGCTTTTCATCATGAGACAG-3′, PGC-1*α*: 5′-GGGATGGCAACTTCAGTAAT-3′ and 5′-AAGAGCAAGAAGGCGACACA-3′, NRF1: 5′-GGGGAACAG AACAGGAAACA-3′ and 5′-CCGTAATGCACGGCTAAGTT-3′, NRF2: 5′-GGGGAACAGAACAGGAAACA-3′ and 5′-CCGTAATGCACGGCTAAGTT-3′, and 18S: 5′-CGAACGTCTGCC CTATCAACTT-3′ and 5′-CTTGGATGTGGTAGCCGTTTCT-3′. The mRNA expression levels were quantified using the 2^-*ΔΔ*CT^ method.

#### 2.5.6. Mitochondrial DNA (mtDNA) Copy Number

Total DNA was obtained from the brain and spinal cord tissue using a commercial kit (Pars Tous, Iran). The mitochondrial DNA copy number was assessed using real-time PCR according to mtDNA (RNR2)/nDNA (GAPDH). Primer sequences were as follows: RNR2: 5′-AGCTATTAATGGTTCGTTTGT-3′ and 5′-AGGAGGCTCCATTTCTCTTGT-3′, and nuclear-encoded GAPDH: 5′-GGAAAG ACAGGTGTTTTGCA-3′ and 5′-AGGTCAGAGTGAGCAGGACA-3′.

### 2.6. Statistical Analysis

The data are presented as mean ± SD. One-way ANOVA with Tukey's test was used for multiple comparisons among groups. The significant level was set as *p* < 0.05. All the calculations were carried out with the GraphPad Prism 5.0 software.

## 3. Results

### 3.1. Behavioural Tests

#### 3.1.1. Passive Avoidance Test (Shuttle Box)

**Figure 1:**
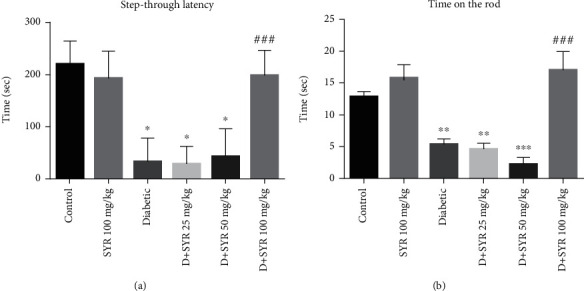
The effects of SYR on the behaviour of streptozotocin- (STZ-) induced diabetic rats in various experimental groups: (a) step-through latency in the shuttle box test and (b) time on the rod in the rotarod test. All data are expressed as mean ± SD; *n* = 6 per group. ^∗^*p* < 0.05, ^∗∗^*p* < 0.01, and ^∗∗∗^*p* < 0.001 vs. the control group; ^###^*p* < 0.001 vs. the diabetic group.

Statistically significant learning and memory deficits were observed in the diabetic group in comparison with the nondiabetic control group (*p* < 0.05). However, no significant difference was observed in the STL of rats treated with 100 mg/kg SYR and rats in the control group. The STL decreased significantly by the administration of 100 mg/kg SYR (*p* < 0.001) in comparison with the STL in the diabetic rats. However, there was no significant difference in STL between the groups treated with 25 and 50 mg/kg of SYR and the diabetic group (Figure 1(a)).

#### 3.1.2. Motor Performance Test

As can be seen in Figure 1(b), in the diabetic group, the delay time in falling from the rotating axis significantly reduced compared to that in the control group (*p* < 0.01). There was no difference in the rotarod performance between the group which was treated with 100 mg/kg SYR and the control group. The time on the rod significantly increased by the administration of 100 mg/kg SYR (*p* < 0.001) as compared with that of the diabetic group.

### 3.2. Biochemical and Molecular Tests

#### 3.2.1. Effect of SYR on Brain GSH Content and AChE Activity

**Figure 2:**
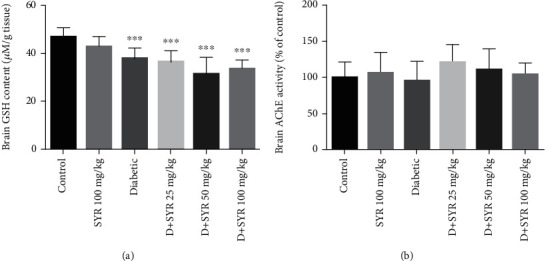
The effects of SYR on (a) brain GSH content and (b) AChE activity of streptozotocin- (STZ-) induced diabetic rats in various experimental groups. All data are expressed as mean ± SD; *n* = 6 per group. ^∗∗∗^*p* < 0.001 vs. the control group.

The GSH content of the brain reduced in all the diabetic groups. Administration of SYR (25, 50, and 100 mg/kg) in diabetic rats did not show any significant changes in GSH levels compared with that of the control diabetic group (Figure 2(a)). The results of AChE activity in the brain tissue are presented in Figure 2(b). No significant differences were observed between the control and diabetic groups or diabetic and SYR-treated groups.

#### 3.2.2. The Effect of SYR on the Level of Lipid Peroxidation, CAT, and SOD Activity

**Figure 3:**
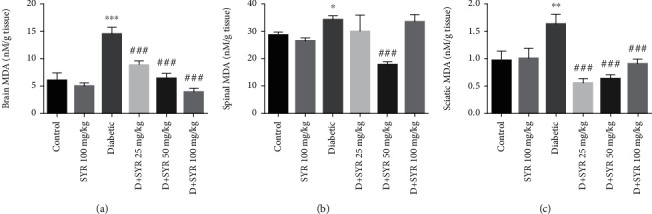
The effects of SYR on lipid peroxidation of the (a) brain, (b) spinal cord, and (c) sciatic nerve of streptozotocin- (STZ-) induced diabetic rats in various experimental groups. All data are expressed as mean ± SD; *n* = 6 per group. ^∗^*p* < 0.05, ^∗∗^*p* < 0.01, and ^∗∗∗^*p* < 0.001 vs. the control group; ^###^*p* < 0.001 vs. the diabetic group.

Significant increases were observed in the level of lipid peroxidation in the brain, sciatic nerve, and spinal cord of the diabetic group, whereas the administration of SYR at 25, 50, and 100 mg/kg reduced MDA level significantly in the brain and sciatic nerve (*p* < 0.001) (Figures 3(a) and 3(c)). While 50 mg/kg was the only dose that was able to reduce the level of MDA in the spinal cord (Figure 3(b)), there were no significant changes in the CAT and SOD activities of all tissues between control and diabetic groups and also among treated groups (data not shown).

#### 3.2.3. The Effect of SYR on Mitochondrial Biogenesis and Mitochondrial Mass

**Figure 4:**
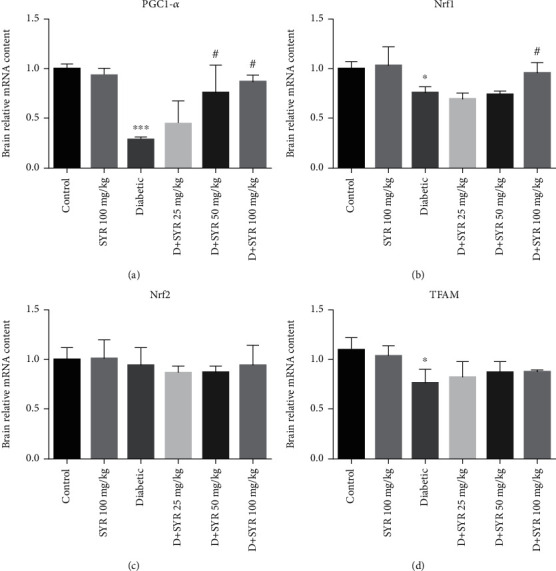
The effect of SYR on mitochondrial biogenesis and mRNA expression in the brain of streptozotocin- (STZ-) induced diabetic rats in various experimental groups. The mRNA expression was measured by real-time RT-PCR. SYR: syringic acid. All values are expressed as mean ± SD; *n* = 4 per group. ^∗^*p* < 0.05, ^∗∗∗^*p* < 0.001 vs. the control group; ^#^*p* < 0.05 vs. the diabetic group.

**Figure 5:**
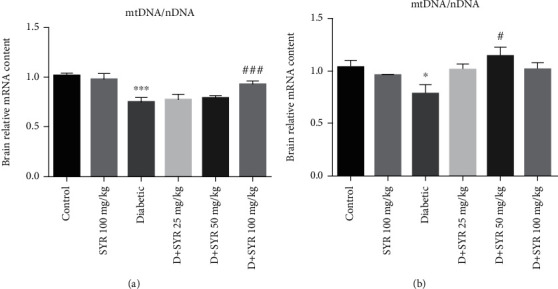
The effect of SYR on mitochondrial mass (mtDNA/nDNA) in the brain and spinal cord of streptozotocin- (STZ-) induced diabetic rats in various experimental groups. SYR: syringic acid. All values are expressed as mean ± SD; *n* = 4 per group. ^∗^*p* < 0.05, ^∗∗∗^*p* < 0.001 vs. the control group; ^#^*p* < 0.05, ^###^*p* < 0.001 vs. the diabetic group.

The mRNA expression levels of PCG-1*α*, NRF-1, NRF-2, and TFAM in the brain were analyzed, as these were indicators of the mitochondrial biogenesis indices. The mRNA levels of PGC-1*α* (*p* < 0.001), NRF-1, and TFAM (*p* < 0.05) were decreased in the diabetic group in comparison with the control group. Administration of 100 mg/kg SYR in diabetic rats increased the mRNA levels of PGC-1*α* and NRF-1 significantly in comparison with those of the diabetic group (*p* < 0.05) (Figures 4(a) and 4(b)). However, this trend was not observed in the TFAM and NRF-2 analyses (Figures 4(c) and 4(d)). Moreover, the analysis of PCG-1*α*, NRF-1, NRF-2, and TFAM showed that there was no significant difference between the control group and 100 mg/kg SYR-treated rats. The amount of mtDNA/nDNA in all groups was measured in the brain and spinal cord tissues since the increase in the mtDNA copy number was considered the index of mitochondrial biogenesis.  Figure 5 shows a significant reduction in the mitochondrial copy number in the diabetic group in the brain and spinal cord tissues as compared with that of the control (*p* < 0.001 and *p* < 0.05, respectively). Furthermore, analysis of the brain exhibited that the mitochondrial copy number in the diabetic group treated with 100 mg/kg SYR increased significantly in comparison with that in the diabetic nontreated subjects (*p* < 0.001). Furthermore, in the spinal cord, there was a significant difference between mtDNA/nDNA in the diabetic subjects and diabetic subjects treated with 50 mg/kg SYR (*p* < 0.05).

### 3.3. Effect of SYR on Pathological Analysis of the Sciatic Nerve

**Figure 6:**
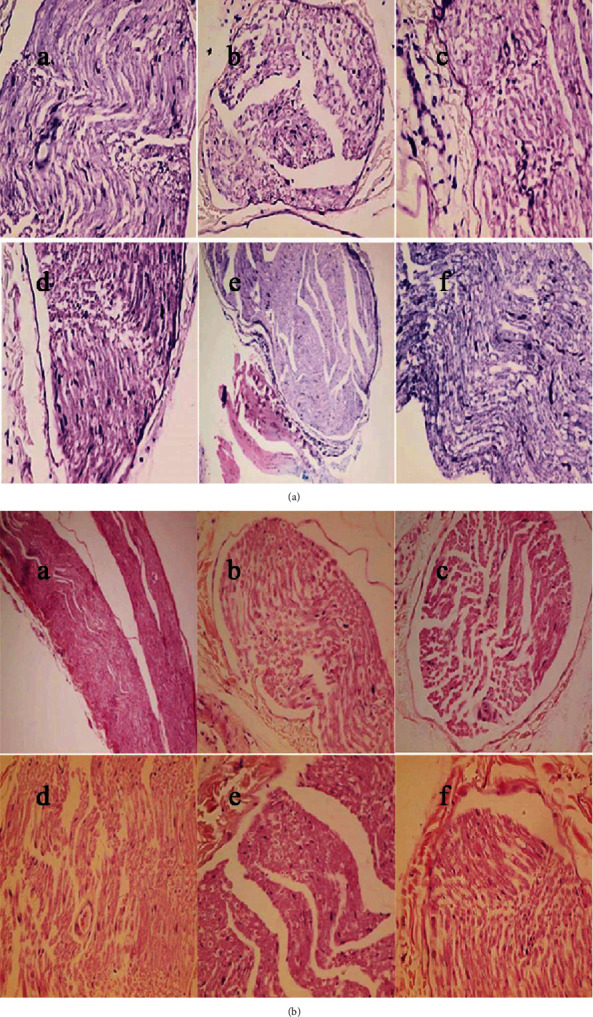
The effect of syringic acid on the histological result of the sciatic nerve of streptozotocin- (STZ-) induced diabetic rats in various experimental groups. (a) Control. (b) Syringic acid (SYR 100 mg/kg), the result is the same as the control normal. (c) STZ group shows inflammation, demyelination, and edema. (d) STZ+SYR 25 mg/kg. (e) STZ+SYR 50 mg/kg groups ameliorate inflammation. (f) STZ+SYR 100 mg/kg group shows the absence of demyelination and inflammatory cells. (a–c) LFB and (d–f) hematoxylin and eosin were used for staining (magnification: 400x).

Pathological analysis using H&E and LFB staining of the sciatic nerve showed inflammation, demyelination, and edema in the diabetic group. In groups receiving SYR (25 and 50 mg/ml), demyelination and edema were seen, but inflammation was ameliorated in comparison with that of the diabetic group. While edema is the only pathological damage in the diabetic group receiving 100 mg/kg SYR, inflammation and demyelination were improved (Figure 6).

## 4. Discussion

The present study investigated the possibility of developing SYR as a potential therapeutic agent for the treatment of diabetic neuropathy (DN). According to the previous studies [15, 21, 31, 32] and also our studies on the protective effects of SYR in the liver and kidney, three safe and effective doses of SYR (25, 50, and 100 mg/kg) were chosen [18, 19]. Results in our previous studies showed an antihyperglycemic effect of 100 mg/kg SYR, which is the critical goal in the control of diabetes (data not shown) [18, 19]. SYR enhances insulin levels by increasing its release from the pancreas and regenerated *β*-cells, restores insulin sensitivity, and increases the consumption of glucose by peripheral tissues [16]. In addition, it is proposed that phenolic acids regulate postprandial glycemia and reduce glucose intolerance by facilitating insulin response and stimulating glucose-dependent insulinotropic polypeptide release and glucagon-like peptide-1 (GLP-1) [33].

Hyperglycemia reduces the antioxidant defence system capacity followed by raising the levels of oxidative stress [34]. DN is a frequent complication of diabetes mellitus that is characterized by spontaneous pain, allodynia, and hyperalgesia [35]. The nervous system is severely sensitive to ROS-related adverse effects because of the high level of polyunsaturated lipids and oxygen consumption, in addition to the lower antioxidant capacity [36]. Furthermore, Zhao et al. found that STZ causes diabetic neuropathy in rats, accompanied by serious oxidative stress in the spinal cord. They reported that lipid peroxidation increased and antioxidant deficiency was observed in the spinal dorsal root ganglia and sciatic nerve in diabetic mice [37]. Thus, the proper antidiabetic factors should be aimed at reducing hypoglycemia as well as improving antioxidant properties.

Our data indicated that the MDA level in the brain, sciatic nerve, and spinal cord increased in the diabetic groups, and administration of SYR significantly reduced the MDA level in these tissues. This finding suggests that SYR has antioxidant properties and ameliorates oxidative damage in STZ-induced diabetic rats.

Furthermore, oxidative stress and reduction of antioxidants play an important role in the impairment of cognitive functions and interruption of short-term spatial memory [38]. Also, previous studies investigated the ameliorating effects of natural compounds including phenolic acids and carotenoids, such as caffeic acid and crocin, on memory, cognitive function, and brain oxidative stress [39–41].

Our results showed that administration of 100 mg/kg SYR significantly decreased step-through latency and increased the remaining time of rats on the rod, so SYR ameliorated the memory loss and motor function in the diabetic rats significantly. Consistent with our study, Ogut et al. reported that SYR is able to improve learning dysfunction and memory suppression by reducing oxidative stress and apoptosis induced by subchronic deltamethrin exposure [42].

Tokmak et al. reported that SYR decreases oxidative damage, apoptosis, and neurodegeneration on pyramidal neurons. They suggested that the neuroprotective effects of SYR are related to reduction of oxidative stress, glial activation, and apoptosis [17]. Other phenolic compounds such as rosmarinic acid [43], epigallocatechin-3-gallate [44], gallic acid [45], and ellagic acid [46] showed positive effects on memory impairment, and glutathione (GSH) is a part of antioxidant defence systems which regulate and maintain the cellular redox status [47]. According to our results, GSH content decreased, while CAT and SOD activities did not change in the diabetic group. However, SYR administration did not have any effect on GSH levels or CAT and SOD activities in the experimental groups, which might be associated with subchronic oxidative stress in the brain, leading to cellular loss of ability to synthesize GSH at a constant level. As SYR is able to scavenge free radicals, it might prevent changes to SOD and CAT activity in the brain and spinal cord of diabetic groups.

Furthermore, according to the previous results, hyperglycemia can cause memory impairment by inducing cholinergic dysfunction. Therefore, the management of cholinesterase activity in addition to the control of hyperglycemia is a factor to consider in diabetes-induced neurodegeneration [48]. In this study, there are no significant differences between AChE in the brain of the diabetic control group in comparison with groups treated with SYR. Thus, SYR may not have a modulatory effect on AChE activity in diabetic conditions, while other phenolic acids (chlorogenic acid) reduced AChE in the hippocampus of the rats with cognitive deficits induced by intracerebroventricular STZ [49].

Besides protein and lipid oxidation, mtDNA is the other target of oxidative damage and reduction of mtDNA is an important factor in diabetes pathogenesis [50]. However, the neuroprotective mechanisms of mitochondrial biogenesis and its association with diabetic neuropathy are not well known. The current approach suggests that increased mitochondrial biogenesis allows the cells to accommodate increased energy loads that promote the cellular viability. Mitochondrial biogenesis enhances the mitochondrial mass, and mitochondrial fission increases the number of mitochondria [51]. Furthermore, oxidative stress induces mtDNA damage in neurons, which is associated with the development of DN. Additionally, the reduction of mitochondrial biogenesis could be the result of insulin resistance [52]. Conversely, mitochondrial regeneration and biogenesis would be a vital process to decrease the risk of DN. Therefore, increasing the number of mitochondria can be a potential strategy in the treatment of DN [53].

The data from this study showed significant decreases in the mtDNA/nDNA ratio in the brain and spinal cord of the diabetic rats. Following the administration of SYR (100 and 50 mg/kg, respectively), the copy number of mitochondria in the brain and spinal cord increased. It is proposed that SYR balanced the mitochondrial homeostasis by increasing mitochondrial biogenesis in these tissues. Increasing mitochondrial biogenesis is associated with a rising mtDNA copy number and SYR-induced mitochondrial gene expression. The gene expression that is involved in this process includes peroxisome proliferator-activated receptor-*γ* coactivator 1 alpha (PGC-1*α*), mitochondrial transcription factor A (TFAM), nuclear respiratory factors 1 and 2 (NRF1 and NRF2, respectively), and mitochondrial transcription factor B1 [54]. Consistent with the aforementioned, DNA microarray analysis showed that PGC-1*α* expression could result in metabolic disorders such as diabetes and insulin resistance [55]. Our results suggest that SYR has the potential to enhance PGC-1*α* and NRF-2 mRNA expression in the brain, two main factors of the transcriptional system in the mitochondrial biogenesis.

The results of the current study showed that mRNA expression levels of PGC-1*α*, NRF-1, and TFAM significantly reduced in the brain of the diabetic rats. Moreover, a significant decrease in the mtDNA/nDNA ratio was observed in the brain and spinal cord of diabetic rats; however, the mitochondrial copy number increased in two tissues following the administration of SYR (100 and 50 mg/kg, respectively). According to the results of previous studies, hyperglycemia leads to an alteration of the PGC-1*α* activator in different cell types. Damaging PGC-1*α* signalling leads to impaired TFAM and NRF2 expression and drives mitochondrial dysfunction [56]. Subsequently, suppression of this pathway plays a critical role in various neurodegenerative disorders such as Alzheimer's, Parkinson's, ALS, and Huntington's disease [56]. Additionally, gene array studies found a reduction in the gene expression of PGC-1*α* and NRF-1 in the skeletal muscle of the patients with diabetes mellitus [57]. Moreover, DN was more serious in diabetic PGC-1*α*-knockout mice [58]. Moreover, in another study, it was observed that coumaric acid upregulated gene expression of NRF1 to initiate mitochondrial biogenesis as an adaptive response in sciatic nerve ischemia-reperfusion injury [59].

According to our results, demyelination, inflammation, and edema were observed in the sciatic nerve of the diabetic group. Inflammatory response can be a consequence of oxidative stress [60]. Treatment of rats with SYR (25 and 50 mg/ml) reduced the inflammation response in the sciatic nerve. Furthermore, SYR treatment (100 mg/kg) reduced demyelination and inflammation in the sciatic nerves.

Under normal conditions, the blood-nerve barrier prohibits the circulating T cells to contact the nerve proteins, but hyperglycemia can damage the nervous vascular barrier. Additionally, glycosylated myelin acts as an antigen and is vulnerable to phagocytic attack [61]. Furthermore, some cytokines such as TNF-*α* and IL-1*β* showed toxic effects on neurons and glial cells, leading to demyelination. On the other hand, advanced glycation end products (AGEs) can increase macrophage phagocytosis of the nerve myelin [61]. Thus, decreasing sciatic nerve demyelination by SYR not only is related to the antihyperglycemic effects of phenolic acids [33] but also can be associated with anti-inflammatory effects, especially to reduce the secretion of TNF-*α*, IL-1*β*, and IL-6 [62]. Future work will focus on the evaluation of the effects of SYR on the protein expression level of mitochondrial genes, as well as the ATP content of brain and spinal cord tissues to further elucidate the mechanism of SYR's therapeutic properties for DN.

## 5. Conclusions

In conclusion, the findings of this study suggest that administration of SYR effectively attenuated oxidative stress and mitochondrial biogenesis in the experimental model of DN.

These modulatory effects of SYR were mediated by preventing lipid peroxidation and scavenging free radicals. Moreover, increasing mRNA expression levels of PGC-1*α*, NRF-1, and TFAM, as well as improving mitochondrial mass of the brain, gives insight into a new mechanism of SYR in the nervous system. Consequently, these findings would encourage future studies on the natural phenolic compounds to reveal other molecular mechanisms in the diabetic neuropathy.

## Data Availability

The data used to support the findings of this study are available from the corresponding author upon request.
